# Iron Overload in Sickle Cell Disease

**DOI:** 10.1155/2010/272940

**Published:** 2010-05-17

**Authors:** Radha Raghupathy, Deepa Manwani, Jane A. Little

**Affiliations:** ^1^Department of Hematology, Albert Einstein College of Medicine and Montefiore Medical Center, Bronx, NY 10461, USA; ^2^Division of Pediatric Hematology/Oncology, Department of Pediatrics, Albert Einstein College of Medicine and Children's Hospital at Montefiore, Bronx, NY 10461, USA

## Abstract

In sickle cell disease transfusions improve blood flow by reducing the proportion of red cells capable of forming sickle hemoglobin polymer. This limits hemolysis and the endothelial damage that result from high proportions of sickle polymer-containing red cells. Additionally, transfusions are used to increase blood oxygen carrying capacity in sickle cell patients with severe chronic anemia or with severe anemic episodes. Transfusion is well-defined as prophylaxis (stroke) and as therapy (acute chest syndrome and stroke) for major complications of sickle cell disease and has been instituted, based on less conclusive data, for a range of additional complications, such as priapism, vaso-occlusive crises, leg ulcers, pulmonary hypertension, and during complicated pregnancies. The major and unavoidable complication of transfusions in sickle cell disease is iron overload. This paper provides an overview of normal iron metabolism, iron overload in transfused patients with sickle cell disease, patterns of end organ damage, diagnosis, treatment, and prevention of iron overload.

## 1. Introduction

The human body has no effective physiological mechanism for excreting excess iron. Therefore, in conditions such as sickle cell disease (SCD), where transfusions are frequently indicated, exogenous iron can accumulate, circulate as nontransferrin bound iron (NTBI), enter tissues, form reactive oxygen species (ROS), and result in end organ damage. However, patients with SCD, compared with thalassemic patients, despite a similar transfusion load, may be relatively protected from iron mediated cardiac and endocrine gland toxicity [1]. In thalassemia, ineffective erythropoiesis contributes to iron overload directly and by regulating other downstream pathways. The unfolding pathophysiology of transfusional iron toxicity in SCD, less well studied than in thalassemia, will be discussed.

## 2. Normal Iron Metabolism

Iron homeostasis in humans is maintained by the strict regulation of absorption based on body needs. 1 mg (10% of total dietary iron) is absorbed daily, predominantly in the duodenum, and an equal amount is lost through feces, urine, and sweat [2]. In normal physiological conditions, iron deficiency and anemia increase iron absorption, while iron overload decreases it [3].

**Figure 1:**
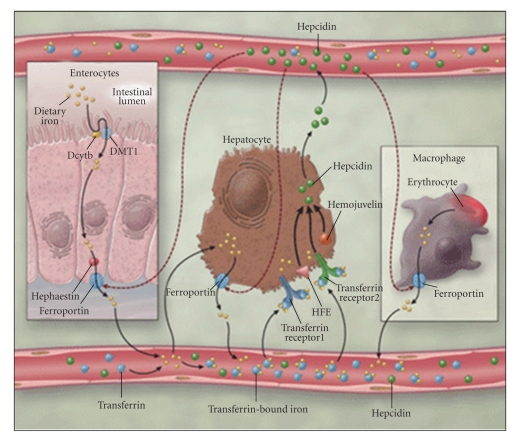
Iron absorption and transport [4]. Reproduced with permission from MMS, and author. Copyright © (2005) Massachusetts Medical Society. All rights reserved.

Nonheme iron absorption is relatively well characterized. Ferric (Fe^3+^) is reduced to ferrous (Fe^2+^) iron in the duodenal enterocyte by a ferric reductase (DcytB). Fe^2+^ is transported into the cell by the Divalent Metal Transporter (DMT-1), located at the apical brush border. In the absence of iron overload, some absorbed iron is stored in the enterocyte as ferritin and the rest is transported across the basolateral membrane by ferroportin, with the aid of the ferroxidase hephaestin. In the circulation, iron is bound to transferrin and transported to the liver and bone marrow. In the liver, transferrin receptors 1 and 2 mediate the endocytosis of iron, which is then stored as ferritin and released by a ferroportin-mediated mechanism when bodily needs increase. In the erythroid precursors, transferrin bound iron is taken up by transferrin receptor 1 and utilized for erythropoiesis. During red cell senescence, iron is released into macrophages in the reticuloendothelial system (RES) and is stored as ferritin and hemosiderin; again, egress of iron from the macrophage is ferroportin dependent [4] (Figure 1).

 The presence of ferroportin on the cell membrane is regulated by hepcidin, a 25-amino acid peptide synthesized by the liver that is the principal hormone involved in regulating iron absorption [5]. Hepcidin acts by binding to the ferroportin transporter, triggering its internalization and degradation, thereby diminishing net circulating iron by reducing iron absorption in the gut and increasing iron sequestration in the RES. Anemia, hypoxia, and erythropoiesis decrease hepcidin gene expression, thereby stabilizing ferroportin and increasing circulating iron available for erythropoiesis [6]. In contrast, acute and chronic inflammation increase hepcidin expression and ferroportin degradation [7]. The paradoxical iron restriction seen in the anemia of chronic inflammation is associated with increased RES iron and results from a high-hepcidin state. Hemojuvelin, also expressed in the liver, is believed to positively regulate hepcidin production [8]. Matriptase 2, a recently identified serine protease, appears to be a sensor of iron deficiency and inhibitor of hepcidin [9]. These peptides help regulate iron absorption and maintain homeostasis. 

Heme iron absorption is less clearly characterized. Heme Carrier Protein 1 (HCP-1), believed to facilitate heme iron uptake, has been recently identified on the duodenal enterocyte brush border [10]. Heme iron taken up by this transporter is broken down by a heme oxygenase in the enterocyte into iron and protoporphyrin [11]. It is unclear whether heme is completely degraded into iron in the enterocyte and absorbed via ferroportin, or if intact heme is also transported via other mechanisms. The Feline Leukemia Virus subgroup C Receptor (FLVCR) appears to play such a role in transporting heme from erythroid precursors [12, 13].

## 3. Indications for Transfusion in SCD

Transfusion is a frequently employed therapy in SCD, but its best-validated uses have been in preoperative prophylaxis, treatment of acute chest syndrome (ACS) and prophylaxis, and treatment of stroke [14–16].

Transfusions first demonstrated their effectiveness in reducing recurrent strokes in SCD [14, 17]. Subsequently, transfusions have also proved to be effective prophylaxis against first stroke in high risk patients. The Stroke Prevention Trial in SCD (STOP) randomized 130 high-risk children with SCD to either transfusion therapy (to maintain HbS <30%) or observation [18]. These high risk children had an increased blood flow in the Internal Carotid or Middle Cerebral Artery by Transcranial Doppler (TCD). This study showed a 92% reduction in incidence of first stroke in transfused high-risk patients. A follow-up study, STOP2, randomized 72 children whose TCD had normalized after 30 months of transfusion therapy to either ongoing or discontinued transfusions. The study was halted prematurely when a significant increase in abnormal TCD velocity and stroke risk was seen in the group in which transfusion therapy had been halted [19]. The optimal duration of transfusion in SCD patients at high risk for primary or recurrent stroke is still undefined, resulting in long-term transfusion management of a young population.

In addition to preventing strokes, transfusion is beneficial in other complications of SCD such as ACS [20, 21]. Vichinsky et al. showed that transfusion improves oxygenation in ACS [22]. Subset analysis from the STOP trial also showed a significant reduction in the frequency of ACS and painful crises in the transfused group [23]. In addition, preemptive transfusion therapy was effective in preventing ACS in a small number of SCD patients with elevated plasma secretory phospholipase A2 levels, an early sign of ACS [24]. In a study of pregnant women (*n* = 72) with SCD randomized to receive prophylactic transfusions or transfusions for medical and obstetric emergencies only, a significant reduction in pain crises was observed in the arm that received prophylactic transfusions [25]. Finally, preoperative simple transfusion to maintain a hemoglobin of 10 gm/dL reduces peri- and postoperative complications in patients with SCD [26–28].

## 4. Mechanism of Transfusion Mediated Iron Overload

While transfusion may improve disease complications, iron overload is a dreaded and inevitable consequence of ongoing transfusion therapy. Chronically transfused iron overloaded patients with SCD have significantly higher mortality than less transfused counterparts without iron overload, as well as age and race-matched normal controls [29, 30]. This finding may reflect disease severity rather than iron burden; nonetheless, patients with SCD are far less likely to be screened for end organ damage than patients with thalassemia, despite a similar transfusion history [31]. Therefore knowledge of iron toxicity in SCD is of paramount importance.

Transfusion of packed red blood cells (RBCs) provides 1 mg per mL transfused of additional elemental iron. Long-term transfusion therapy of, for instance, 20-units RBCs/year is associated with significant iron overload (20 units × 220 mL per unit × 1 mg per mL = 4400 mgm exogenous iron/year) [32]. With repeated transfusions, serum transferrin becomes saturated and the excess circulating iron is transported as NTBI [33]. NTBI enters cells in a dysregulated fashion; a subset of NTBI, called Labile Plasma Iron (LPI), may cause end organ damage secondary to its high redox potential [34].

## 5. End Organ Toxicity due to Iron Overload in SCD

**Figure 2:**
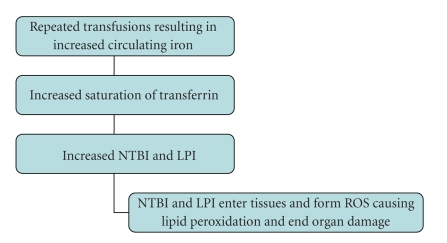
Mechanism of end organ damage in iron overload.

Saturation of transferrin by excess circulating iron results in increased NTBI and LPI [35]. NTBI can appear in the absence of transferrin saturation, the mechanism of which is not yet clear [36]. NTBI and LPI tend to enter tissues more readily and result in formation of reactive oxygen species (ROS) such as the hydroxyl radical by the Haber Weiss reaction [37, 38]. Excess iron tends to deposit in the hepatic parenchyma, in endocrine organs, and in cardiac myocytes, causing end organ damage by ROS-mediated lipid peroxidation [39, 40] (Figure 2).

In patients with *β* thalassemia major (TM), long-term transfusion causes iron overload that results in cardiac damage, liver fibrosis, gonadal dysfunction, and growth retardation. Cardiac iron overload still remains the main cause of mortality in TM despite chelation therapy [41]. 

Although large studies looking at long-term transfusion and iron overload in SCD are lacking, available data suggest that SCD patients are relatively protected from iron-induced cardiac and endocrine organ damage as compared with TM patients. In a study by Wood et al. comparing 19 patients with TM and 17 patients with SCD (matched for age, sex, transfusion duration, chelation therapy, and hepatic iron content), cardiac iron overload, measured by T2*MRI, and cardiac dysfunction were significantly more prevalent in the group with TM [42]. In another study, cardiac disease and gonadal dysfunction seen in TM were not present in SCD patients with similar serum ferritin levels and equivalent liver iron content. Hepatic fibrosis was detectable pathologically, albeit at 39% in SCD (*n* = 43) compared with 81% in TM (*n* = 30). TM patients had a significantly longer duration of transfusions and higher incidence of viral hepatitis in this study [43].

Studies comparing the sequelae of iron overload in TM versus SCD are of interest, but one must be cautious in applying conclusions from these studies given the many differences between TM and SCD patients clinically. These reports highlight the need for independent study of the contribution of iron overload to the morbidity and mortality in SCD.

## 6. Potential Factors Modifying Iron Toxicity in SCD

The possible difference in iron mediated toxicity between TM and SCD underscores the complexity of iron regulation. Damage from iron overload is not merely a function of the absolute amount of excess iron present. Modifying factors include, but are not limited to, relative distribution of iron loading in RES versus parenchymal cells, levels of NTBI and LPI, coexisting hereditary iron loading defects, and the impact of ineffective erythropoiesis. 

The levels of NTBI and LPI, the most redox reactive and toxic iron species, have been compared between SCD and TM. In a small study, NTBI levels in SCD were less than half of those seen in TM, despite higher ferritin levels and hepatic iron concentration in SCD. However, patients with thalassemia in this study had received 4 times more transfusion than those with SCD [44]. Koren studied patients with SCD including HbSS and S*β*° thalassemia (*n* = 36) and *β*  Thalassemia, including TM and Thalassemia intermedia (*n* = 43). The two groups overall were matched for transfusion load (SCD: 104 ± 77 and *β*  Thalassemia 179 ± 103 cc/kg/yr). In subset analyses, however, TM patients had a significantly greater transfusion burden than patients with HbSS, which may account for significantly lower NTBI and LPI observed in SCD patients in this study. While only 1 patient with SCD developed cardiomyopathy, 32 patients with TM had evidence of either cardiac or gonadal damage [45].

Coexistent hereditary iron overload conditions have been studied in the context of SCD and the iron overload phenotype. Currently defined hemochromatosis polymorphisms of European origin are less frequent in African Americans and do not seem to contribute to exacerbated iron loading in African Americans with SCD [46]. Nonetheless, other causes of primary and dietary iron overload are well described in populations of African descent, even if the underlying genetics are not, and these may contribute to phenotypic diversity in SCD and iron overload [47, 48].

The role of hepcidin in the pathophysiology of iron overload in SCD remains controversial. Patients with SCD suffer from recurrent infections and ongoing endothelial damage by reperfusion injury that result in a chronic-inflammatory-like state [49]. This is evidenced by increased CRP, IFN*γ*, IL-1, and IL-6 in steady state and IL-6 and TNF-*α* increase during crises [50, 51]. By analogy with the anemia of chronic inflammation (in which cytokines increase hepcidin levels), one may speculate that hepcidin levels would be elevated in SCD. However, hepcidin levels in nontransfused steady-state SCD (*n* = 40) are not elevated when compared with normal controls (*n* = 30), even when SCD patients with infections and end organ damage were assessed as a subgroup [52]. Another study, comparing hepcidin in 16 patients with sickle syndromes (HbSS, HbS*β*° thalassemia, HbSC) against race matched controls heterozygous for HbS or HbC, also found no difference among the groups. In fact, in 5 patients with SCD in this study, hepcidin levels were below the lower limit of normal, which was attributed to increased erythropoietic activity [53]. Coexistent iron deficiency can also affect hepcidin estimation. Iron deficiency anemia has been described in the pediatric population with SCD, both due to nutritional status and intravascular hemolysis with urinary iron losses. In a study of 70 children with HbSS and HbSC, 9% of nontransfused children were found to have iron deficiency [54]. 

Although steady-state SCD patients do not exhibit increased hepcidin levels, transfusion acutely up regulates hepcidin mRNA expression [55]. It is unclear whether this increased gene expression results in increased circulating hepcidin and decreased unbound iron immediately post transfusion. Nor is it known whether this transient increase in hepcidin expression is of clinical relevance. 

In contrast, hepcidin is significantly downregulated in patients with ineffective erythropoiesis such as thalassemia, thereby worsening unbound iron toxicity in this population [56–58]. Depressed urinary hepcidin levels relative to iron burden have been associated with augmented but ineffective erythropoiesis (thalassemia, congenital dyserythropoietic anemia and sideroblastic anemia). This is in distinction to conditions with augmented but effective erythropoiesis, as in hemolysis (hereditary spherocytosis, and autoimmune hemolytic anemias), in which urinary hepcidin levels relative to iron burden are not significantly depressed [59]. Hepcidin suppression in thalassemia may be mediated in part by growth differentiation factor 15 (GDF-15). GDF-15, a member of the TGF-*β* superfamily, is produced by erythroid progenitors and reaches measurable serum levels in the presence of ineffective erythropoiesis. GDF-15 levels are elevated in TM, but not in SCD. Serum from TM patients suppresses hepcidin expression in vitro, unless GDF-15 is blocked [60]. Twisted gastrulation (TWSG1), a cytokine produced during early stages of erythropoiesis, also downregulates hepcidin and is highly expressed in the spleen, bone marrow, and liver of thalassemic mice. The role of TWSG1 in SCD has not been studied yet [61]. Hepcidin and other cytokines (such as GDF 15 and TWSG1) are likely to be key to observed iron regulation in SCD.

## 7. Measurement of Iron Overload

The gold standard for assessing liver iron stores in the absence of cirrhosis is the hepatic iron content (HIC), determined by liver biopsy and quantitation with atomic absorption spectrophotometry [62]. The normal HIC is between 0.4 and 2.2 mg/g of liver dry weight. Based on data from hereditary hemochromatosis, <7 mg/g is not associated with obvious hepatic pathology while >15 mg/g is consistently associated with liver fibrosis [63]. The use of biopsy-measured HIC as a marker of iron overload is limited by the small but finite risk of complications of liver biopsy, lack of reproducibility of quantitative assays, and sampling error [64].

Noninvasive methods including blood tests (ferritin and iron saturation) and imaging techniques (MRI-based techniques) have been evaluated as predictors of HIC. Ferritin has been shown to correlate with HIC in TM, but the correlation in SCD is less clear [65]. In a cross-sectional study of 27 children with SCD who had received chronic transfusion therapy without chelation, transfusion volume correlated with hepatic iron content (HIC) but, when adjusted for transfusion volume, serum markers did not [66]. In another study of 20 patients with SCD undergoing chronic transfusion therapy with iron chelation, HIC showed a positive correlation with the duration of transfusion and liver fibrosis but not with serum markers [67]. Analyses of chronically transfused SCD patients without viral hepatitis from the STOP and STOP2 trials, most of whom were on chelation therapy, showed that a ferritin level <1500 ng/mL was correlated with low transfusion burden and low measured HIC, while a ferritin >3000 ng/mL was consistently predictive of HIC >10 mg/g. The intermediate levels did not correlate linearly with iron overload [68]. These data suggest that serum ferritin may not be an accurate predictor of liver iron stores, especially in the range of 1500 to 3000 ng/mL. 

Imaging techniques for noninvasive determination of HIC have been developed and validated. The Superconducting Quantum Interference Device (SQUID) quantitatively determines HIC by magnetic measurement, and in cases of hereditary and transfusional iron overload has shown significant correlation with HIC as measured by biopsy [69]. However, this device is expensive and not readily available. T2* MRI has been validated as a reliable noninvasive means to assess iron stores in the liver and heart [70]. Increasing iron content in the liver and heart reduces relaxation times as measured by T1, T2, and T2* MRI. T2* values of <20 milliseconds in nonfibrotic livers correlates (*r* = 0.93) with increased HIC by biopsy [71]. Similarly, cardiac T2* values of <20 ms correlate with a decline in left ventricular ejection fraction values, increase in cardiac arrhythmias, and need for cardiac medications [72]. The reciprocal of this relaxation time, R2 and R2*, has also demonstrated good direct correlation with HIC in patients with SCD [73]. From these data it appears that MRI is a good noninvasive tool to assess iron overload (Table 1).

## 8. Therapy for Transfusional Iron Overload in SCD

### 8.1. Non Pharmacological Therapy

Decreasing transfusion requirement by substituting erythracytapharesis for simple transfusion, transfusing younger red cells, and performing splenectomy in patients with hypersplenism have been evaluated as nonpharmacological techniques for preventing and treating iron overload. In erythrocytapharesis, patient RBCs are removed as transfused blood is being infused. A study was performed using erythrocytapharesis to a goal HbS of 50% in patients with history of stroke, recurrent VOC or priapism, comparing them to historic controls treated with simple transfusion or modified simple transfusions. The degree of iron overload, as assessed by serum ferritin, was significantly reduced in 14 SCD patients treated by erythrocytapharesis compare to those treated with simple transfusion, in spite of an increased absolute requirement for donor units [74]. This approach is hampered by practical resource limitations at donor centers and by complications of permanent vascular access in SCD [75, 76]. Furthermore, increased exposure to donor units also raises the risk of alloimmunization to further deter its wider acceptance in the community [77].

Transfusion of young erythrocytes called neocytes significantly reduces transfusion requirements and prolongs transfusion interval by 15–25%. However, this occurs at the expense of increased donor exposure and costs [78], and this technique is not widely used.

Retrospective analysis of 34 pediatric patients with SCD (HbSS, HbSC, HbS*β*° thal) for 6 months before and 12 months after splenectomy for splenic sequestration or hypersplenism showed a significant reduction in transfusion requirements postsplenectomy [79]. Splenectomy, however, is associated with long-term risk of infection by encapsulated organisms and likely benefits few adult patients with SCD since autosplenectomy commonly leaves them without a functioning spleen.

### 8.2. Pharmacological Therapy

Chelation therapy is routinely employed to prevent and treat iron overload in chronically transfused SCD patients. Chelators work by targeting the unbound iron in the blood, including NTBI and LPI that cause tissue injury [80]. Different parameters have been used to estimate iron load and determine need for chelation. These include HIC by liver biopsy, serum markers such as ferritin, and transfusional iron load (TIL).

#### 8.2.1. Indications to Commence Chelation Therapy

Traditionally, determining HIC by liver biopsy is considered the gold standard to predict iron overload and determine need for chelation therapy. Based on data from hereditary hemochromatosis and TM showing that elevated HIC over 7 mg/g liver dry weight is a risk factor for hepatic fibrosis, this value has been used as a guide to start chelation [63, 81]. In a study of 12 pediatric patients with SCD who previously received an average of 15 transfusions over 21 months, mean HIC was 9.4 ± 1.2 mg/g dry weight, and 4 of 12 patients had liver fibrosis, validating that a cut off of 7 mg/g could be used as a guide for SCD as well [82]. Vichinsky et al. described 43 adult patients with SCD who were previously transfused for a mean of 6 years, resulting in elevated ferritin levels at 2916 ± 233 ng/mL, and increased HIC, at 14.33 ± 1.4 mg/g dry weight. Only 2% of these SCD patients had viral hepatitis, but 39% of them had an elevated fibrosis score at liver biopsy suggesting that adults with SCD have a similar response to iron overload [43].

As mentioned previously, noninvasive estimates of HIC are less predictive of the need for liver biopsy and possible therapy for iron overload. Serum ferritin of >1000 ng/mL, used as a guide in patients with thalassemia, is not validated in SCD. While in SCD ferritin levels of over 3000 ng/mL are associated with HIC >10 mg/g, values between 1500 and 3000 ng/mL are not predictive of an elevated HIC [67]. A better parameter for noninvasive estimation is transfusional iron load (TIL). TIL of >100 mg/kg has been closely correlated with high liver iron stores and liver fibrosis in the pediatric population and is an indication to start chelation therapy [65, 67] (Table 2). There are no systematic studies in adults with SCD. 30 units of RBCs in a 70 Kg adult would result in 94 mg/Kg iron load, and so findings in children with SCD are congruent with recommendations for secondary iron overload in myelodysplastic syndrome in adults, where chelation is suggested after transfusion of 20–30 units of packed RBCs, or a ferritin of >2500 ng/mL [83] (see National Cancer Comprehensive Network Clinical Practice Guidelines in Oncology v.2.2010) (http://www.nccn.org/professionals/physician_gls/PDF/mds.pdf).

#### 8.2.2. Chelating Agents

Deferoxamine (DFO), the first iron chelator introduced in the 1970s, is derived from the microbe *Streptomyces pilosus*. Due to poor oral absorption it is administered parenterally. In the liver it is converted to active metabolites that chelate-free iron and eliminate it through urine and feces. Only 10% of the administered drug is available for chelation [84]. Although initially given intramuscularly, therapy was later optimized as subcutaneous infusions of 30–50 mg/kg for 8–12 hours every night for 5–7 nights per week [41]. Dose adjustment is performed based on ferritin to DFO ratio. With good compliance the drug prevents and reverses cardiac dysfunction and significantly improves survival [85]. Adverse effects include reversible sensorineural hearing loss, retinal damage, and growth retardation [86]. Yearly audiologic and ophthalmologic evaluations are advised since early effects are reversible. Rare cases of renal failure and interstitial pneumonitis have also been reported. 

However, continuous parenteral administration makes DFO less attractive, and compliance is poor [87, 88]. Children and adults with thalassemia have been treated with subcutaneous DFO injections every twelve hours as a more acceptable alternative to continuous infusions, though the amount of drug that can be administered by bolus injections is limited [89, 90].

Oral alternatives include Deferasirox (DFS) and Deferiprone (DFP). DFS is an FDA approved oral iron chelator. In a randomized open label multicenter phase II study, one year of DFS therapy was compared against DFO in chronically transfused SCD and both drugs were found to be equally efficacious. Side effect profile of DFS was similar to DFO including >10% headache, skin rash, and gastrointestinal toxicity as well as less common hearing and visual side effects [91]. Renal toxicity has been described in DFS, and the target dose for efficacy is approximately 25–30 mg/Kg. Compliance is variable because of taste, and DFS, like DFO, is expensive.

Deferiprone is an oral iron chelator licensed for use in Europe. Safety of this drug was demonstrated in a multicenter prospective study but long-term data regarding efficacy is lacking. It appears to have the greatest efficacy in chelating cardiac iron but, because of early controversial studies in North America, is not licensed for use by the FDA [92, 93].

The efficacy and safety of combination therapy using different chelators is also being studied.

## 9. Conclusion

Iron overload is a feared complication of long-term transfusion in SCD. The indications for transfusion in SCD continue to broaden and minimizing unnecessary transfusions in patients with SCD should be strongly emphasized in the clinical setting to reduce complications of iron overload. Optimum use of nontransfusion therapy such as Hydroxyurea may mitigate some, but not all need for transfusion therapy. Whether nonmyeloablative bone marrow transplantation replaces long-term transfusion therapy in some situations remains to be seen. SCD patients may be relatively protected from end organ damage due to iron toxicity, for reasons incompletely understood. Hepcidin is likely central to differences observed between iron toxicity in SCD and in thalassemia.

In SCD, a condition with a strong inflammatory component, ferritin, which is an acute-phase reactant, may not accurately predict body iron stores. Based on currently available data (often extrapolated from other diseases), chelation therapy should be considered when:

1. adult patients with SCD have received 20–30 units of RBC transfusions,
2. pediatric patients with SCD are approaching a TIL ≥ 100 mg/Kg, and/or
3. HIC in any age group exceeds 7–9 mg/g
  - Excessive HIC is likely when the serum ferritin is >3000 ng/mL, less clear at 1500–3000 ng/mL.

Oral and parenteral chelators with a range of side effects and costs are now available that can be tailored to individual patients; nonetheless, simple and inexpensive chelation remains an elusive goal.

Challenges remain in the prevention and management of iron overload in SCD. Increased federal research support would benefit clinical investigation into practical matters such as clarification of the indications for transfusion therapy in adults with SCD, improved catheter technology to allow safe exchange transfusions, and optimal chelation therapy. Important unaddressed pathophysiologic questions include the etiology of nonhepatic risks of iron overload in adults (such as pulmonary hypertension, the risk for which has been correlated with ferritin levels), and a more complete elucidation of the role that perturbed iron absorption, transport, and storage play in damage from iron overload in SCD.

## Figures and Tables

**Table 1:** Tests to estimate iron load.

TEST	Application
Serum ferritin	Relatively unreliable in SCD, especially in the range of 1500–3000 ng/mL. In this range, levels of ferritin do not linearly correlate with HIC

SQUID	Reliable predictor of HIC, but expensive and available in few institutions worldwide, mostly for research purposes

T2* MRI	Well-validated predictor of HIC and cardiac complications from iron overload

Liver biopsy	Gold standard. Accurate estimation of iron overload except in fibrosis. Invasive but <1% risk of complications. Sampling error possible

**Table 2:** Indications for chelation therapy in SCD.

Test	Adult patients	Pediatric patients
Transfusional iron load	20 to 30 units	>100 mg/kg
Serum ferritin	>3000 ng/mL, 1500–3000 ng/mL: equivocal	>3000 ng/mL, 1500–3000 ng/mL: equivocal
HIC	>7–9 mg/g dry weight	>7–9 mg/g dry weight
